# Decreased Ovarian Reserves With an Increasing Number of Previous Early Miscarriages: A Retrospective Analysis

**DOI:** 10.3389/fendo.2022.859332

**Published:** 2022-06-10

**Authors:** Jifan Tan, Lu Luo, Jiaxin Jiang, Niwei Yan, Qiong Wang

**Affiliations:** ^1^ Reproductive Medicine Center, The First Affiliated Hospital, Sun Yat-sen University, Guangzhou, China; ^2^ Guangdong Provincial Key Laboratory of Reproductive Medicine, Guangzhou, China

**Keywords:** previous miscarriages, ovarian reserve, anti-Müllerian hormone, antral follicle count, recurrent miscarriage

## Abstract

The fact of ovarian reserve (OR) decreased in women with recurrent miscarriage has been well known. However, Whether OR would decrease with increasing numbers of previous miscarriages (PMs) is still unclear. To address this, OR parameters of following four groups’ patients were evaluated: 99 women with one previous miscarriage (PM1), 46 women with two previous miscarriages (PM2) and 35 women with three or more previous miscarriages (PM3). The control group included 213 women without a history of miscarriage (PM0). The correlation of OR parameters and the proportion of diminished ovarian reserve (DOR) patients between the four groups were analyzed using Kendall’s Tau-B coefficients. The results showed the median anti-Müllerian hormone (AMH) levels were 4.04, 3.40, 3.14 and 2.55 respectively in the PM0, PM1, PM2 and PM3 groups, respectively (H=15.99, *P* = 0.001); the median antral follicle counts (AFCs) were 10, 8, 8 and 6, respectively (H=24.53, *P* < 0.001); and the proportions of DOR patients were 10.8%, 15.2%, 23.9% and 31.4% (χ2 = 13.01, *P* = 0.005). In addition, AMH level and AFC correlated negatively with the number of PMs (correlation coefficients -0.154, *P* < 0.001; -0.205, *P* < 0.001 respectively), the proportion of DOR patients correlated positively with the number of PMs (correlation coefficients 0.156, *P =* 0.001). After stratification by age, AMH and AFC levels were still significantly lower in the PM3 group than the PM0 group (*P* < 0.05). The proportion of DOR patients between the PM0 and PM3 groups was statistically significant (*P* < 0.001). This study showed that AMH levels and AFCs decreased as well as the proportion of DOR patients increased significantly as the number of PMs increased. In conclusion, our study indicates decreased AMH levels and AFCs might be one of the factors contributing to early miscarriage.

## Introduction

Miscarriage is defined as spontaneous loss of a pregnancy before 22 weeks of gestational age (1), which is recognized as a relatively common event, occurring in 15-20% of pregnancies. Most miscarriages are early, occur before 12 weeks of gestational age (1, 2).

Approximately 1–2% of the women face three or more consecutive spontaneous miscarriages with the same partner, termed recurrent miscarriage (RM). Even after a detailed evaluation of the etiology of early miscarriage, 50% of RM cases remained unexplained, idiopathic, or unknown (1–3).

Abnormal or low-quality oocytes could be a potential factor contributing spontaneous pregnancy losses in these women (4, 5). Ovarian reserve (OR) demonstrates reproductive potential as the number and quality of remaining oocytes. The basal serum levels of basal follicle-stimulating hormone (FSH), luteinizing hormone (LH), estradiol (E_2_), and FSH : LH ratio, inhibit B, anti-Müllerian hormone (AMH) and antral follicle count (AFC) are parameters of OR, which have been proven to be predictors of oocyte quantity (6–10). Although still be controversial, they are also thought to be possible predictors of oocyte quantity (9–12). Diminished ovarian reserve (DOR) is a common phrase for evaluating the value of OR, which is important to provide the clinical counseling for women with the previous miscarriages (PMs) (4, 9, 13, 14).

Recent studies have documented an association between RM and decreased OR, using AMH levels and AFCs as markers of OR (9, 14, 15). In addition, many of these studies showed that the proportion of DOR patients in the RM group was significantly higher than that in the control group (9, 14). However, whether OR would decrease with the number of PMs is still unclear. As an approach to address this problem, we conducted a cohort study to explore the association between OR parameters and the proportion of DOR patients with increasing numbers of PMs.

## Materials and Methods

### Patients

All patients who had undergone assisted reproductive technology (ART) therapy because of female pelvic or fallopian tube factor infertility at the Center for Reproductive Medicine of the First Affiliated Hospital of Sun Yat-sen University from October 2016 to May 2018 were retrospectively included. The study was performed after receiving approval from the Medical Ethics Committee of the First Affiliated Hospital of Sun Yat-sen University (No. 2016115), and informed consent was obtained from all patients.

All patients were classified into the following study groups according to their medical records: 99 women with one previous early miscarriage (PM1), 46 with two previous early miscarriages (PM2) and 35 with three or more previous early miscarriages (PM3). Additionally, the control group included 213 women without a history of miscarriage (PM0). All patients underwent routine investigations of infertility and examinations to exclude factors known to interfere with OR, including parental chromosomal abnormalities, a history of ovarian surgery, endometriosis, structural uterine abnormalities (e.g., unicornuate uterus, uterine septum) and endocrine abnormalities (e.g., thyroid dysfunction, polycystic ovary syndrome). Women in the PM2 and PM3 groups were further investigated to exclude possibly known causes of miscarriage, including autoimmune disease, antiphospholipid syndrome, lipidemia, hemolysis and thrombophilia. The exclusion criterion was abnormal results during any of the aforementioned investigations or hemolysis. Finally, 393 women who underwent IVF-ET because of pelvic or fallopian tubal factors met the eligibility criteria.

### Measurements of Ovarian Reserve Parameters

We used the following six OR tests: AMH, AFC, follicle-stimulating hormone FSH, LH, E_2_, and FSH : LH ratio. The serum levels of FSH, LH and E2 were measured during the early follicular phase (Days 3-5) of the menstrual cycle within 6 months before the initiation of IVF cycles. An ultrasound assessment of the number of AFCs measuring 2–10 mm in diameter between the second and the fifth days of the menstrual cycle was performed by a single skilled physician who was blinded to the patients’ conditions. All women involved in the study were administered a long-term or short-term gonadotropin releasing hormone agonist (GnRH-a) for pituitary downregulation during the mid-luteal phase, according to the protocol. Before initiating gonadotropin injections, blood samples were collected from a peripheral vein on the 3rd to the 5th day of a menstrual cycle and then centrifuged at 3000 r/min for 15 mins at room temperature. Then the serum was separated and stored in a refrigerator at –80°C.

The serum levels of FSH (normal range: 2.00-138.00 IU/L), LH (normal range: 0.40-105.00 IU/L) and E2 (normal range: 0.00-300.00 pg/ml) were measured using the chemiluminescence method (Architect Alinity, Abbott, Longford, Ireland). The serum level of AMH (normal range: 0.24-11.78 ng/ml) was measured using an enzyme-linked immunosorbent assay (ELISA) (Diagnostic Kits for the Quantitative Detection of AMH; GK Biological Technology Ltd).

### Statistical Analyses

The Kolmogorov–Smirnov test was used to assess the distribution of data. Nonnormally distributed data were analyzed using the Kruskal–Wallis test for comparisons between multiple groups and the Mann–Whitney U test for comparisons of two groups. The chi-square test was used to compare categorical data and Bonferroni comparisons were performed for two groups. Kendall’s Tau-B correlation coefficients were calculated to describe the correlations between variables. Significance was defined as a *P* value <0.05.

## Results

### The Baseline Characteristics and Variables Indicating of the OR Between Groups

The baseline characteristics and variables indicating of the OR are presented in **Table 1**. The mean age of the 393 included women was 33.86 years (range: 25–45 years). The numbers of PMs ranged from 0 to 10. The mean age, proportions of women aged <35 years and ≥35 years and body mass index (BMI) values did not differ significantly among the groups (P>0.05).

**Table 1:** Comparison of baseline characteristics and parameters of the ovarian reserve between groups.

Variables	PM 0 (n = 213)	PM 1(n = 99)	PM 2 (n = 46)	PM 3 (n = 35)	*P*-value
<35 years	138 (64.00%)	49 (49.49%)	25 (54.35%)	19 (54.29%)	0.061
≥35 years	75 (35.21%)	50 (50.51%)	21 (45.65%)	16 (45.71%)	
BMI (kg/m2)	20.83 (19.48, 23.01)	21.08 (19.29, 23.23)	21.01 (19.42, 23.46)	21.64 (20.03, 24.03)	0.340
AMH (ng/ml)	4.04 (2.32, 6.93)	3.40 (2.05, 5.06)	3.14 (1.36, 6.10)	2.55 (1.30, 3.78)	0.001
AFC (number)	10 (7, 15)	8 (6, 12)	8 (6, 10)	6 (5, 10)	<0.001
FSH (IU/L)	5.55 (4.67, 6.67)	5.79 (4.79, 6.96)	5.20 (4.53, 6.92)	5.83 (4.52, 7.58)	0.641
E2 (pg/ml)	34.00 (26.25, 45.75)	36.00 (27.00, 44.00)	35.00 (27.00, 46.00)	36.00 (29.00, 55.00)	0.636
LH (IU/L)	3.48 (2.66, 4.45)	3.21 (2.49, 4.18)	3.58 (2.25, 4.35)	3.28 (2.58, 4.16)	0.745
FSH/LH(value)	1.63 (1.21, 2.27)	1.75 (1.38, 2.35)	1.77 (1.33, 2.28)	1.74 (1.21,2.60)	0.598

Nonnormally distributed data were presented as numbers (%) or as medians (interquartile ranges).

OR, ovarian reserve; PM0, no previous miscarriages; PM1, one previous miscarriage; PM2, two previous miscarriages; PM3, three or more previous miscarriages; BMI, body mass index; AMH, anti-Müllerian hormone; AFC, antrum follicle count; FSH, follicle-stimulating hormone; E2, estradiol; LH, luteinizing hormone.

### The Relationship Between AMH Levels and AFCs Numbers With an Increasing Number of PMs

Regarding the variables indicating of OR (**Table 1**), the median AMH levels were 4.04, 3.40, 3.14 and 2.55 in the PM0, PM1, PM2 and PM3 groups, respectively, and the differences were statistically significant (H= 15.99, P = 0.001). The median AFCs values were 10, 8, 8 and 6 in the PM0, PM1, PM2 and PM3 groups, respectively, and the differences were statistically significant (H= 24.53, *P* < 0.001). According to the Mann–Whitney U test for comparisons of pairs of groups, the differences in the AMH level and AFC between the PM0 and PM3 groups were statistically significant (*P* < 0.001), while the differences between other groups were not statistically significant. In contrast, the comparisons of basal FSH and E2 levels and FSH/LH ratios did not yield significant differences.

### The Correlations Between the PM Number and Age With OR

**Figure 1:**
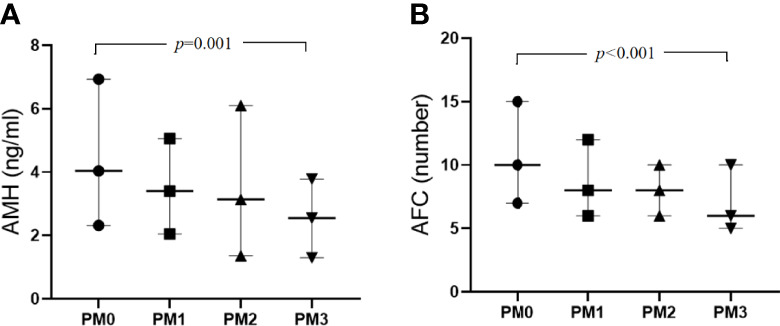
Comparisons of AMH level **(A)** and AFC **(B)** numbers show a decreasing trend from PM0 to PM3.

Regarding the variables indicative of OR, AMH and AFC showed a decreasing trend (**Figure 1**). Kendall’s Tau-B correlation was subsequently used in this study to evaluate the correlations between OR markers (AMH concentration, AFC), and age with the number of PMs (**Table 2**). The AMH level, as a marker of OR function, exhibited significant negative correlations with both age and the number of PMs (Kendall’s Tau-B_the numbers of PMs_= -0.154, *P <* 0.001; Kendall’s Tau-B_age_=-0.340, *P* < 0.001). Similar results were also observed when the AFC was used as a marker of OR function. Age and the number of PMs were negatively correlated with AFC (Kendall’s Tau-B _the numbers of PMs_= -0.205, *P* < 0.001; Kendall’s Tau-B_age_=-0.339, *P* < 0.001). The comparison of other OR functions (basic FSH and E2 levels and the FSH/LH ratio) showed no significant differences.

**Table 2:** Correlation analyses of PM number and age with AMH levels and AFCs levels (by Kendall’s Tau-B correlation).

parameters	AMH		AFC	
	correlation coefficient	*P* -value	correlation coefficient	*P-* value
Age	-0.340	<0.001	-0.339	<0.001
Number of PM	-0.154	0.001	-0.205	<0.001

PM, previous miscarriage; AMH, anti-Müllerian hormone; AFC, antral follicle count.

### The Relationship Between the PM0 and PM3 Groups and OR

As maternal age was identified as a prominent confounding factor affecting AMH levels and AFC, we further compared the PM0 and PM3 groups after stratification by age (**Table 3**). Among participants aged <35 years, AMH and AFC levels were both significantly higher in the PM0 group (median 4.96 ng/ml, IQR [2.99-8.00] and median 11 number, IQR [8-15], respectively) than in the PM3 group (median 3.62 ng/ml, IQR [1.71-5.49], *P* = 0.01 and median 6 number, IQR [5-8.5], respectively; *P* < 0.05). Among women aged >35 years, the AMH level was also significantly higher in the PM0 group than in the PM3 group (median, [IQR]: 2.81[1.48-4.82] *vs*. 2.00[1.19-2.82], *P* = 0.03); however, the AFCs did not differ significantly (median, [IQR]: 8[6-11.75] *vs*. 6[4-10.5], *P* = 0.25). The AFC was still lower in the PM3 groups than in the PM0 groups but was not different.

**Table 3:** Comparisons of parameters of OR between the PM0 and PM3 groups with age stratification.

Age	<35years			≥35years		
parameters	PM 0	PM 3	*p*-value	PM 0	PM 3	*p*-value
Age (years)	30.49±2.39	31.26±2.53	0.19	38.13±2.73	39.19±2.54	0.16
AMH (ng/ml)	4.78 (2.96,8.35)	3.70 (1.76.5.37)	0.01	2.81 (1.48,4.82)	2.00 (1.13,2.96)	0.03
AFC (ug/L)	11.00 (8.00,15.00)	6.00 (5.00,8.50)	<0.001	8.00 (6.00,11.50)	6.00 (4.00,10.50)	0.25
FSH (ug/L)	5.42 (4.55.6.39)	5.06 (4.27,7.02)	0.50	5.86 (5.00,7.43)	7.27 (5.37,7.73)	0.14
E2 (ug/L)	34.00 (26.75,44.00)	36.00 (27.00,55.00)	0.29	34.50 (25.75,48.00)	36.50 (29.00,59.50)	0.52
LH (ug/L)	3.59 (2.68,4.49)	3.39 (2.87,4.30)	0.86	3.35 (2.51,4.45)	2.72 (2.53,4.16)	0.71
FSH/LH (value)	1.53 (1.13,2.09)	1.51 (0.95,2.39)	0.78	1.88 (1.45,2.42)	2.23 (1.52,2.81)	0.32

Data are presented as medians (interquartile ranges).

OR, ovarian reserve; PM0, no previous miscarriages; PM3, three or more previous miscarriages; AMH, anti-Müllerian hormone; AFC, antrum follicle count; FSH, follicle-stimulating hormone; E2, estradiol; LH, luteinizing hormone.

### The Relationship and Correlations Between the PM Number and Age With DOR

Thus far, the definition of DOR remains controversial. In the present study, the DOR was defined as an FSH level ≥10 mIU/mL and/or AMH level < 1.1 ng/mL and/or AFC< 5 by referring to a large number of studies and conference reports (9, 14–16). Using chi-square values from PM0 patients, the proportions of DOR were 10.8%, 15.2%, 23.9% and 31.4% in the groups PM0, PM1, PM2 and PM3 groups, respectively, with statistically significant differences (χ2 = 13.01, *P* = 0.005). According to the Bonferroni pairwise comparison, the difference in the proportion of DOR patients between the PM0 and PM3 groups was statistically significant (*P*<0.001), while the comparisons between other groups showed no significant differences (**Table 4**). In addition, the proportion of DOR patients showed significant positive correlations with both age and the number of PMs (Kendall’s Tau-B _the numbers of PMs_ = 0.156, *P* = 0.001; Kendall’s Tau-B_age_=0.277, *P* < 0.001) (**Table 5**).

**Table 4:** Comparison of the proportion of DOR patients among groups.

	PM0	PM1	PM2	PM3	*p*
DOR	23 (10.8%)	15 (15.2%)	11 (23.9%)	11 (31.4%)	0.005
Non-DOR	190 (89.2%)	84 (84.8%)	35 (76.1%)	24 (68.6%)	

PM, previous miscarriage; DOR, diminished ovarian reserve.

**Table 5:** Correlation analyses of PM number and age with DOR (by Kendall’s Tau-B).

parameters	DOR	
	correlation coefficient	*p-* value
Age	0.277	<0.001
Number of PM	0.156	0.001

PM, previous miscarriage; DOR, diminished ovarian reserve.

## Discussion

Our study revealed a significant negative correlation between the number of PMs and OR after adjusting for maternal age, as measured by AMH levels and AFCs. To the best of our knowledge, the present study is the first to report a possible relationship between OR parameters and the proportion of DOR patients with the number of PMs. Previous studies mainly explored the potential association between the OR and RM, not including the PM1 or PM2 groups. We also verified that low AMH levels and AFCs in women with RM in both women aged <35 years and those aged >35 years. In addition, the percentages of women with DOR in the RM group were significantly higher than those in the PM0 group, consistent with recently reported observations (9, 14).

In our study, only the PM3 group had a significantly lower AMH levels and AFCs than the PM0 group. The respective comparisons of the PM0 group with the PM1 and PM2 groups did not yield statistically significant results. In addition, we conducted the Kendall’s Tau-B correlation analyses and confirmed a significant negative correlation of AMH levels and AFCs with the number of PM after adjusting for maternal age. Therefore, in women with advanced maternal age or DOR who are predisposed to a greater risk of more miscarriages, and adverse effects become apparent and only then does OR decrease to a sufficient extent. It is well known that OR are thought to be possible markers of oocyte quality (6, 7). Besides, it is assumed that quality of oocytes could be one of the potential factors related to aberrant embryo quality or embryo aneuploidy which may lead to subsequent early miscarriage (17, 18). Our study also supported the hypothesis that a decreased OR might be a potential contributing factor to the etiology of the PM.

OR represents the quality and quantity of the remaining oocytes. The basal serum levels of basal FSH, E2, LH, FSH/LH, inhibin B and AMH, as well as the AFC, ovarian stromal vascularization and ovary volume, are commonly used to assess OR (4, 13), However, the value of these OR tests to predict the quality and quantity of the ovarian primordial follicular pool remains controversial (9, 14, 18, 19). Hormone levels fluctuate with the menstrual cycle and vary between laboratories to laboratories (19, 20), but they are still used in most units as a baseline test for ovarian function. The present study showed no statistically significant differences in the levels of FSH and E2 between women with RM and PM0, which was consistent with numerous studies (21–23). However, studies by Atasever, et al. (14) and Trout, et al. (24) reported statistically significant differences in the levels of FSH and E_2_ between the RM and explained RM groups. In addition, similar to our study, Tow studies (14, 21) showed no statistically significant differences in the FSH : LH ratio between women with RM compared with the non-RM group.

In contrast, the serum AMH level, a novel marker of OR, is a dimeric glycoprotein produced solely by the ovarian follicles and is not affected by the menstrual cycle. Recently, an increasing number of studies have verified that lower AMH levels in women with RM than in non-RM groups (9, 14, 15, 25); Similarly, our results also showed that women with three or more previous unexplained miscarriages had lower AMH levels. However, one study by Prakash et al. (26) suggested that AMH levels were not significantly different between RM and non-RM groups.

The AFC has a better value in predicting a poor response and OR than the basal FSH level. In the present study, we found that women with RM had lower AFC levels, suggesting an association with decreased OR, but no statistically significant differences were observed between the PM1, PM2 and PM0 groups. A systematic (9) review also supported the hypothesis that low AFCs predict a higher odds of miscarriages. However, Atasever, et al. (14)found that the AFC level in the RM group was not significantly different from that in the non-RM group, but the results may have been affected by difficulties in obtaining an accurate AFC, such as intraobserver variability and anatomic variations.

Accordingly, AMH levels and AFCs are closely related, and they are considered the most reliable and accurate markers of OR (4, 13, 18, 19). Based on our results, these two markers might also be considered to have the best prognostic value. Recently, Sophie Pils et al. (27) confirmed the effect of the number of PMs, which predicted the outcome of further pregnancies in women with RM through a multivariate analysis. In our study, we found that AMH levels and AFCs decreased with increasing numbers of PMs. We suggest that a decreased quantitative OR may predict poor oocyte quality which affects the next pregnancy. Blood samples were analyzed under the same conditions, and previous studies have reported that different ovulation stimulation protocols, including GnRH-a, have no effect on the results of AMH analyses (28). Therefore, we propose that the AMH measurements in our study are stable and reliable and therefore did not affect the final conclusions.

In recent decades, DOR has gradually become a challenge for women of reproductive age and those using reproductive medicine (9, 14, 29). However, the criteria for defining DOR remains controversial, including the selection of various OR functional markers and the specific ranges. AMH levels and AFCs have been widely used OR markers. However, the detected values of AMH levels are quite different because of the diversities of testing methods, laboratory conditions, the absence of international unified standards and quality control systems, and other factors. Similarly, AFC measurements are highly subjective. Research has shown that AMH levels and AFCs combined with FSH levels jointly predict DOR, which is an overall assessment of egg quantity and quality and is conducive to the prediction of embryo quality and pregnancy outcomes (29, 30).

In this study, the DOR patients were defined as an FSH level ≥10 mIU/mL and/or an AMH level <1 ng/mL and/or AFC<5, based on a previous expert consensus, academic conferences and related studies (9, 16, 29–31). In contrast, previous studies reporting on DOR with RM used cutoff values of FSH levels, E_2_ levels, AMH levels and AFC that had not been updated as the definition of DOR. Our study found the similar higher odds of DOR in the RM group, consistent with the aforementioned studies (9, 14, 32). We previously knew that patients with DOR had high miscarriage rates, and many patients with DOR were of advanced age. Thus, for many years, we were unable to differentiate whether high miscarriage rates in patients with DOR were associated with advanced age. Recently, Morin SJ et al. (33) reported that an aneuploidy rate less than 38 in patients with DOR was similar to that in controls, even in patients with higher FSH levels and lower AMH levels and AFCs. In our study, we found that the association of miscarriage women associated with DOR was evenly balanced with age.

Our study explored the number of PM groups and multiple parameters of OR which is in different with previous studies. However, one limitation of present study is that our sample size is still limited, especially in the PM2 and PM3 groups. In future, large prospective cohort studies are warranted and mechanism of OR decreased on spontaneous miscarriage should be explored. Similar to advanced age women, Patients with RM also have high embryo aneuploidy and decreased OR (8, 34, 35), but it is still unknown whether their mechanism was similar or not. The other limitation of present study is the absence of chromosome information in no PM group patients because of its retrospective approach.

In conclusion, our study found that AMH levels and AFCs decreased as well as the proportion of DOR patients increased significantly as the number of PMs increased. Decreased AMH levels and AFCs might be a contributing factor to early miscarriage. We believe that these new insights could be useful in counseling for couples with PM. DOR should be predicted in multiple PMs patients and early interference should be conducted to achieve the better pregnancy outcomes in the future.

## Data Availability Statement

The raw data supporting the conclusions of this article will be made available by the authors, without undue reservation.

## Ethics Statement

The studies involving human participants were reviewed and approved by The First Affiliated Hospital of Sun Yat-sen University on 22 Jun 2016 (No. 2016115). The patients/participants provided their written informed consent to participate in this study.

## Author Contributions

JT and QW contributed to the design of the work. JT and LL contributed to manuscript written and statistical analyses. Data acquisition and analysis were carried out by JJ and NY. JT and LL carried out the statistical analyses. QW critically reviewed the paper. All authors contributed to the article and approved the submitted version.

## Funding

The study was supported by Natural Science Foundation of Guangdong Province (2021A1515010290) and National Natural Science Foundation of China (81871159).

## Conflict of Interest

The authors declare that the research was conducted in the absence of any commercial or financial relationships that could be construed as a potential conflict of interest.

## Publisher’s Note

All claims expressed in this article are solely those of the authors and do not necessarily represent those of their affiliated organizations, or those of the publisher, the editors and the reviewers. Any product that may be evaluated in this article, or claim that may be made by its manufacturer, is not guaranteed or endorsed by the publisher.

## Abbreviations

PM1, one previous miscarriage; PM2, two previous miscarriages; PM3, three or more previous miscarriages; PMs, previous miscarriages; RM, recurrent miscarriage; OR, ovarian reserve; DOR, diminished ovarian reserve; AMH, Serum levels of anti-Mullerian hormone; AFC, antral follicle count; FSH, follicle-stimulating hormone; LH, luteinizing hormone.
